# LONGITUDINAL DYNAMICS OF THE GUT MICROBIOME AND SERUM PROTEOME OVER THE MOUSE LIFESPAN

**DOI:** 10.1093/geroni/igaf122.1798

**Published:** 2025-12-31

**Authors:** Yanjiao Zhou, Yair Dorsett, Ruixuan Li, Kun Chen

## Abstract

In mice, the gut microbiota undergoes dynamic changes from early development through aging. Parallel to these microbial dynamics, the host serum proteome reflects systemic physiological states and responses to microbial and environmental cues. However, longitudinal studies integrating both the gut microbiome and serum proteomics in the same animals remain limited. We collected stool samples from 15 mice every two weeks and blood every three months, from young adulthood until death at around 2–3 years of age. Principal component analysis and DE-SWAN analysis revealed five waves of microbiome changes across the lifespan, with a major compositional shift occurring in middle age. Longitudinal clustering identified four distinct patterns of microbial dynamics. Proteomic analysis of serum markers revealed that different functional groups of host proteins exhibited distinct age-related trajectories. Inflammatory cytokines and chemokines rose sharply in late middle age, whereas central nervous system (CNS)-associated markers increased predominantly in old age. Increases in CNS serum markers correlated most strongly with expansions in Proteobacteria and, even more so, with Cyanobacteria. Such integrated analyses enable the identification of coordinated host–microbe changes, offering a systems-level view of aging biology and potential biomarkers for age-associated dysfunction.

